# The Barber, the Surgeon, and the Taster

**Published:** 1908-10-31

**Authors:** 


### Medical Antiquities.

### The Barber, the Surgeon, and the Taster.

The barber's pole is of interest both on account of its association with the infancy of surgery and because it is the last existing relic of the old trade signs. Its red, white, and blue stripes indicate that the office of barber was combined with that of surgeon, or, to be more precise, that of bleeder.

Until the twelfth century the clergy combined the offices of priest and physician, and cared alike for body and soul. The shedding of blood, however, was deemed unsuited for the priestly craft; therefore at the Council of Tours in 1163 Pope Alexander III. forbade the clergy to act as surgeons.

The barber, who had long assisted the priest, seeing his opportunity, took unto himself the office of phlebotomist. As the craft increased its numbers the members thereof banded themselves into a guild, primarily a social and religious institution, but eventually a trade organisation. In 1308 Richard was sworn as first master of the Barbers' Company, but it was not until 1462 that it obtained a charter. In 1540 the name was changed to that of Barber-surgeons. The artist Holbein's last picture represents Henry VIII. and the Barber-Surgeons, a painting still preserved at the Barber-Surgeons' Hall in Monkswell Street.

As may well be supposed, this encroachment upon the prerogatives of the surgeon proper was not unopposed. Dispute followed dispute, and efforts were made to confine each to his own sphere. An Act of Henry VIII. states: "No person using any shaving or barbery in London shall occupy any surgery, letting of blood, or other matter, except of drawing teeth." But it was easier to make than to enforce the Act, and it was not until 1745 that the surgeons and the barbers were legally and finally separated. The barber-surgeon survived for a long time; the last, a man named Middleditch, of Great Suffolk Street, died as late as 1821.

In 1797 Lord Thurlow stated in a speech to the House of Lords: "By a statute, still in force, barbers and surgeons were each to use a pole. The barbers were to have theirs blue and white, striped, with no other appendage; but the surgeons', which was to be the same in other respects, was likewise to have a gully-pot and a red tag to denote the particular nature of their vocations." The origin of the pole as a trade sign seems to have arisen in the following way. It was usual for the venesector to give his patient a staff to hold, around which, for convenience, was wrapped the bandage. This pole was exhibited in the window or doorway. Later this was represented by a pole painted in a spiral manner with blue and white stripes. Another addition was made by introducing a red stripe, to represent the artery, while the original blue and white represented the vein and bandage respectively. Unfortunately in these later days other colours have been introduced into the decorative scheme of the barber's pole, thus destroying the significance of that quaint emblem.

The crown of a mediæval monarch sat uneasily upon his brow, and not the least of his worries was the ever-present dread of poison. Because of this all food and drink supplied to the king's table was duly sampled beforehand. Usually the office of assayer was filled by a medical man. The preparation of and participation in a meal in the regal household must have been an imposing ceremony. After the covers had been laid, the bread and salt duly tasted, the table linen and cutlery kissed, the whole table was covered by a cloth which remained untouched until the head of the household took his seat. Before the dishes left the kitchen the assayer proceeded thither, and not only partook of samples, but tested the integrity of cook and stewards by compelling them to do the same. Nor was this all, for all the viands were again put to the test at the table itself by the appointed assayer. This ceremony, which took a considerable time, was usually accompanied by music. When entertaining a distinguished guest a special taster was appointed to him.

From Hall's "Chronicle," edition 1548, we learn that at Pontefract Castle Sir Piers Exton, who wished to destroy the ill-fated Richard II., forbade the "esquire whiche was accustomed to serve and take the assaye beefore Kyng Richarde, to again use that manner of service." Further, the King "sat downe to dyner, and was served without curtesie or assaye." We also gather that when the King heard that it was the order of Sir Piers Exton, he struck the assayer on the head with the carving knife, exclaiming "The Devil take Henry of Lancaster and thee together." The drink also was tasted, first by the butler in the buttery and also by the cup-bearer under the eye of the master. A special assay cup was used in which was often embedded a charm believed to have the power of detecting poison.

